# Association between a functional variant in *RAD51* gene’s 3′ untranslated region and its mRNA expression in lymphoblastoid cell lines

**DOI:** 10.1186/s40064-016-3339-2

**Published:** 2016-09-29

**Authors:** Fengxia Chen, Haozhong Zhang, Feifei Pu

**Affiliations:** 1 Department of Medical Oncology, General Hospital of The Yangtze River Shipping, Wuhan, 430022 Hubei People’s Republic of China; 2 Department of Orthopedics, Union Hospital, Tongji Medical College, Huazhong University of Science and Technology, 1277 Jiefang Avenue, Wuhan, 430022 Hubei People’s Republic of China

**Keywords:** Genetic, MicroRNA, Polymorphism, *RAD51*, Variant

## Abstract

**Object:**

Variants of microRNA (miRNA)-binding sites in *RAD51* gene’s 3′ untranslated region (3′UTR) are significantly associated with cancer risk, but the roles of these genetic variants in post-transcriptional regulation have not been elucidated.

**Methods:**

The SNPs of *RAD51* were identified both in the regulatory region and in the coding region by means of the online database. The bioinformatic tool SNP Function Prediction was used to predict the potential functional relevance of the miRNA-binding sites. We used additional data on *RAD51* genotypes and mRNA levels available online for the genotype-phenotype association analysis.

**Results:**

We found that rs12593359, rs7180135, rs11855560, and rs45507396 in the *RAD51* 3′UTR affect possible miRNA-binding sites according to bioinformatic analysis. Only rs12593359 was significantly associated with *RAD51* mRNA expression in lymphoblastoid cell lines (P = 0.022).

**Conclusion:**

This study demonstrated that rs12593359 may be a putative variant mediating the post-transcriptional regulation of the *RAD51* gene. Deeper understanding of how 3′UTR variants influence *RAD51* activity will pave the way to targeting of the *RAD51* pathway as a cancer treatment.

## Background

Cancer is a common fatal disease and results from complex interactions between environmental and genetic factors (Pharoah et al. 2004). More and more studies have been focused on the role of gene polymorphisms in the etiology of cancers. Lately, there is growing evidence that single nucleotide polymorphisms (SNPs) play an important role in carcinogenesis (Zeljic et al. 2014; Vispé et al. 1998). DNA repair systems are believed to maintain genome integrity by countering threats posed by DNA lesions. Deficiencies in DNA repair pathways may leave such lesions unrepaired or repaired incorrectly, eventually leading to genomic instability or mutations that may contribute directly to cancer.

The *RAD51* gene is located in chromosomal region 15q15.1 in humans (Shinohara et al. 1993). The RAD51 protein encoded by *RAD51* is necessary for the repair of DNA damage. Growing evidence indicates that *RAD51* performs an irreplaceable function in the maintenance of genomic stability and in the repair of DNA double-strand breaks (Baumann and West 1998). *RAD51* genetic variations may contribute to the development of cancers (Thacker 2005). Overexpression of *RAD51* was also found to be associated with a poor prognosis in squamous cell carcinoma of the head and neck, colorectal cancer, ovarian cancer, and breast cancer (Gresner et al. 2012; Krupa et al. 2011; Romanowicz-Makowska et al. 2012; Wieqmans et al. 2014). A variety of molecular epidemiological studies have been conducted to estimate the association between the *RAD51* 135G/C polymorphism and risk of cancers (Wang et al. 2013; Cheng et al. 2014). In addition, *RAD51* is intimately involved in both the pathogenesis of malignant tumors and progression of various types of cancer.

It is well known that variants of microRNA (miRNA)-binding sites can alter gene functions. MiRNAs can regulate the activity of *RAD51*, and miRNA dysregulation has been implicated in neoplasms. For instance, Huang and colleagues used luciferase reporter constructs containing the *RAD51* wild-type or mutant 3′UTRs, and they found that miR-103/107 directly targets the 3′UTR of *RAD51* and that miR-103/107 overexpression in combination with cisplatin or other DNA-damaging agents may therefore have therapeutic utility in terms of chemosensitization of tumors (Huang et al. 2013). In addition, a number of studies have shown that *RAD51* variants may perform crucial functions in carcinogenesis, but the role of variants of miRNA-binding sites of *RAD51* is still unknown (Thacker 2005; Gresner et al. 2012). In the present study, we performed a bioinformatic analysis and genotype-phenotype association analysis by means of the HapMap database to test our hypothesis that *RAD51* 3′UTR variants are associated with miRNA regulation of this gene’s expression.

## Methods

### Bioinformatic analysis and selection of polymorphisms

The SNPs of *RAD51* were identified both in the regulatory region and in the coding region by means of the online database (http://www.ncbi.nlm.nih.gov/SNP/). The bioinformatic tool SNP Function Prediction (FuncPred; http://www.snpinfo.niehs.nih.gov/cgi-bin/snpinfo/snpfunc.cgi) was used to predict the potential functional relevance of the miRNA-binding sites. Additionally, we limited our analysis to SNPs with minor allele frequency (MAF) >5 % in the HapMap populations “Utah residents with Northern and Western European ancestry (CEU)” and calculated pairwise linkage disequilibrium (LD) values of all the SNPs in the same gene. Then, we selected the SNPs that were not in LD (r^2^ < 0.8) and constructed LD maps of those SNPs of the *RAD51* gene using a Web service (http://www.snpinfo.niehs.nih.gov/cgi-bin/snpinfo/snpfunc.cgi).

### Genotype data and mRNA expression data on lymphoblastoid cell lines from the HapMap database

We used additional data on *RAD51* genotypes and mRNA levels available online (http://www.app3.titan.uio.no/biotools/help.php?app=snpexp) for the genotype-phenotype association analysis (Holm et al. 2010). We used genome-wide expression arrays (47,294 transcripts) from Epstein–Barr virus-transformed lymphoblastoid cell lines from the same 270 HapMap individuals (142 males and 128 females) to analyze the gene expression variations (Stranger et al. 2007). The data on genotyping were retrieved from the HapMap phase II release 23 dataset consisting of 3.96 million SNP genotypes from 270 individuals from 4 populations (International HapMap Consortium 2003). A tool called SNPexp v1.2 was used for calculating and visualizing the correlations between HapMap genotypes and gene expression levels (Norwegian PSC Research Center, Clinic for Specialized Surgery and Medicine, Oslo University Hospital Rikshospitalet, Norway).

### Ethics statement

The study was approved by the Ethics Committee of Union Hospital Tongji Medical College of Huazhong University of Science and Technology and conforms to the provisions of the Declaration of Helsinki.

### Statistical methods

Genotype-phenotype correlations were analyzed by the χ^2^ test. All statistical tests were two-sided, and data with P < 0.05 were considered statistically significant.

## Results

### Selected variants of *RAD51* 3′UTR and putative miRNA-binding sites

In total, 849 SNPs were identified in the *RAD51* gene region, and 38 in the mRNA-coding region (http://www.ncbi.nlm.nih.gov/SNP/). Among them, 24 SNPs are located in the 3′UTR, and only 4 SNPs of these (rs12593359, rs7180135, rs11855560, rs45507396) have a known MAF value >0.05. These four SNPs were predicted to affect the miRNA-binding site activity according to the bioinformatic analysis, as shown in Table 1. The most extensively studied SNPs of these affect putative binding sites of hsa-miR-129-3p, hsa-miR-1248, hsa-miR-1303, hsa-miR-197, hsa-miR-220b, hsa-miR-767-5p, hsa-miR-876-5p, hsa-miR-642, and hsa-miR-299-5p (http://www.snpinfo.niehs.nih.gov/cgi-bin/snpinfo/snpfunc.cgi). In combination with other SNPs in the 3′UTR or promoter region, variant rs7180135 is jointly involved in cancer susceptibility (Teo et al. 2012).

**Table 1:** Selected SNPs of RAD51 3′UTR and putative miRNA binding sites

Name	Alleles	MAF	Putative miRNA binding sites
rs12593359	G/T	0.416	hsa-miR-129-3p
rs7180135	A/G	0.273	hsa-miR-1248,hsa-miR-1303,hsa-miR-197,hsa-miR-220b,hsa-miR-767-5p,hsa-miR-876-5p
rs11855560	C/T	0.416	hsa-miR-642
rs45507396	A/G	0.086	hsa-miR-299-5p

*SNP* single nucleotide polymorphism; *3′UTR* 3′ untranslated region; *MAF* minor allele frequency; *NA* not available

### Calculation of LD of all SNPs in the *RAD51* gene

**Fig. 1:**
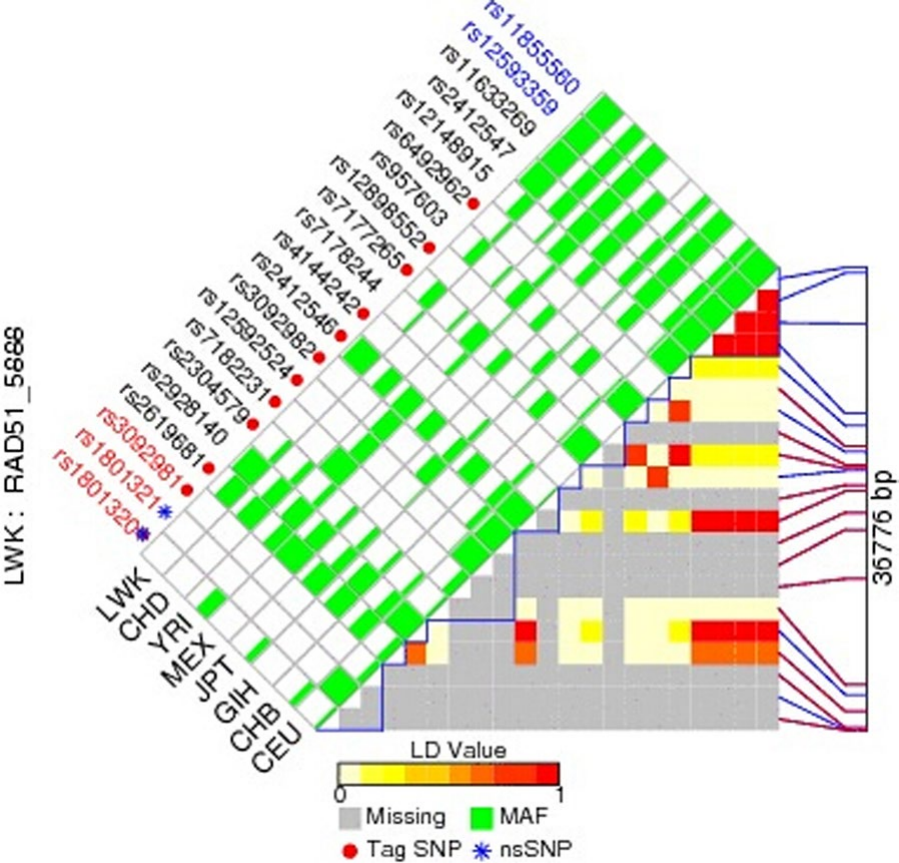
A linkage disequilibrium plot of the RAD51 region according to SNP Function Prediction software (FuncPred). Each *square number* represents the pairwise r^2^ correlations between the two relevant SNPs. The color of each SNP spot reflects its D′ value, which changes from red to white as the D′ value decreases. *SNP* single nucleotide polymorphism

The bioinformatic tool FuncPred (http://www.snpinfo.niehs.nih.gov/snpfunc.htm) helped us to identify the potential functional relevance of the SNPs. We calculated pairwise LD values of all SNPs in the same gene to select the SNPs that were not in LD (r^2^ < 0.8), and plotted the LD maps of those *RAD51* SNPs by means of FuncPred. The pairwise r^2^ correlations between the two relevant SNPs were represented by each square number. The color of each SNP spot reflects its D′ value, and when the D′ value decreases, the color changes from red to white. The haplotype blocks were estimated using the FuncPred software. The MAF of all of the above alleles was greater than 0.05. Polymorphisms rs12593359 and rs11855560 were the predicted tag SNPs in our study, whereas rs7180135 and rs45507396 in *RAD51* were not included in the LD plot (Fig. 1).

### RAD51 mRNA expression by genotypes in lymphoblastoid cell lines

**Fig. 2:**
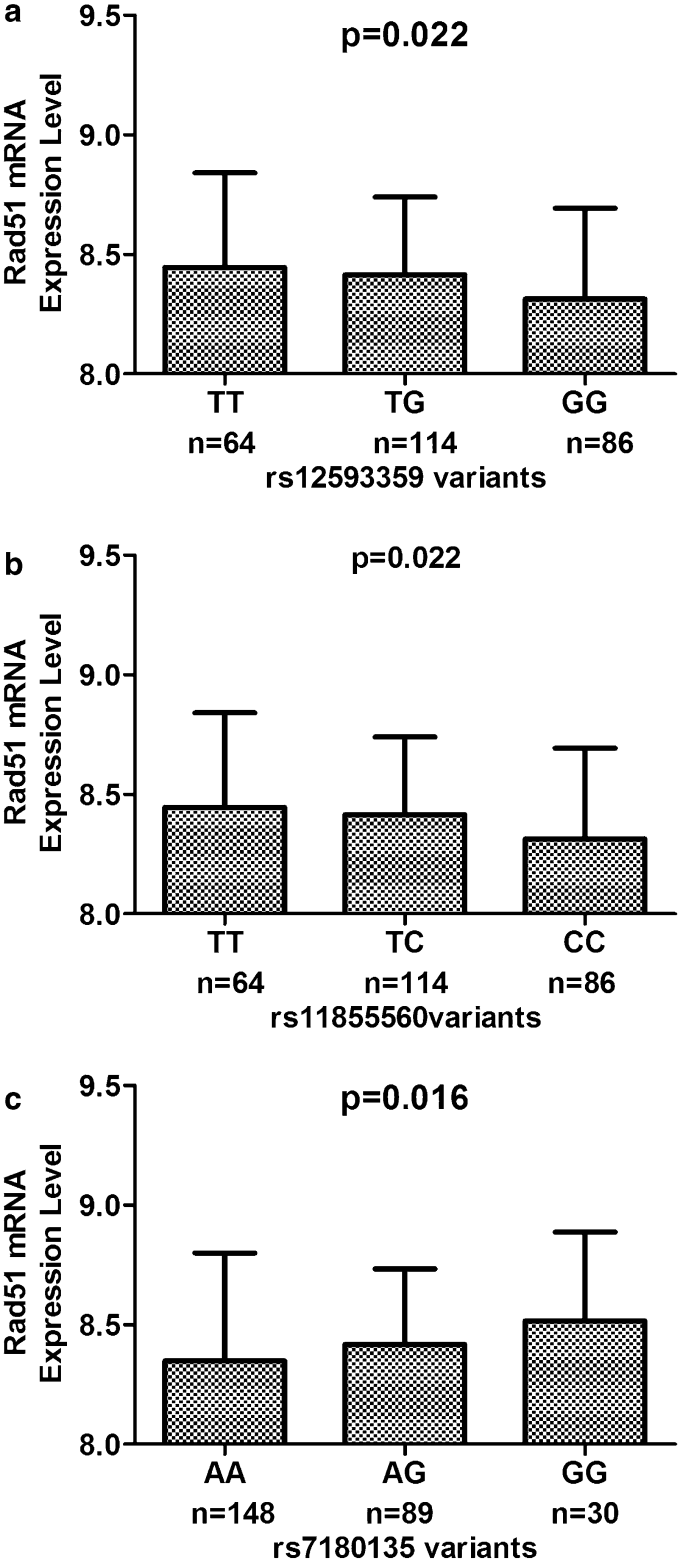
The mRNA expression levels of *RAD51* uncovered by the genotype–phenotype association analysis of *RAD51* variants, rs12593359 (**a**), rs11855560 (**b**), rs7180135 (**c**) and *RAD51* mRNA expression in Epstein-Barr virus-transformed lymphoblastoid cell lines from the HapMap database

For evaluation of mRNA expression of the prohibitin gene in the lymphoblastoid cell lines, we took advantage of the available HapMap-cDNA expression database to analyze the correlation of prohibitin genotype with mRNA expression in 270 HapMap lymphoblastoid cell lines. With the exception of the one cell line with unavailable values for rs12593359, 64 (24.24 %) cell lines with the TT genotype, 114 (43.18 %) cell lines with the TG genotype, and 86 (32.58 %) cell lines with the GG genotype were identified. For rs11855560, 64 (24.24 %) cell lines had the TT genotype, 114 (43.18 %) the TC genotype, and 86 (32.58 %) cell lines had the CC genotype. For rs7180135, 148 (55.43 %) cell lines had the AA genotype, 89 (33.33 %) cell lines the AG genotype, and 30 (11.24 %) cell lines had the GG genotype. Figure 2 shows the *RAD51* mRNA expression levels of cell lines by *RAD51* genotype. The rs12593359 GG genotype was found to yield significantly lower expression levels than the TT and TG genotypes did (P = 0.022; Fig. 2a), and there were significant differences in *RAD51* mRNA expression levels among the cell lines carrying rs11855560 (P = 0.022) and rs7180135 (P = 0.016) genotypes (Fig. 2b, c).

## Discussion

The *RAD51* gene, a homolog of recA from *Escherichia coli*, has been mapped to chromosomal region 15q15.1 in humans (Shinohara et al. 1993). It spans >39 kb, contains ten exons and encodes a 339-amino acid protein (GenBank accession No.: NM_133487). The *RAD51* gene produces a protein also called RAD51, which is necessary for repair of damaged DNA (Lo et al. 2003). A number of studies, such as tissue expression experiments, animal models, and clinical trials suggest that *RAD51* plays an important role in carcinogenesis, and numerous research groups have analyzed the link between *RAD51* polymorphisms and cancer risk (Cheng et al. 2014; Maacke et al. 2000; Vispe et al. 1998). In the present study, the effect of SNPs in genes involved in DNA repair mechanisms on the response to treatment and survival in gastric cancer patients treated with platinum-based chemotherapy was investigated (Ding et al. 2015).

The present study supports the notion that mutations in miRNA-binding regions contribute to altered gene function. Although our findings indicate that rs12593359, rs7180135, rs11855560, and rs45507396 in the *RAD51* 3′UTR affect potential miRNA-binding sites according to bioinformatic analysis, we found that rs12593359 (P = 0.022), rs7180135 (P = 0.022), and rs11855560 (P = 0.016), but not rs45507396, were significantly associated with *RAD51* mRNA expression levels in lymphoblastoid cell lines. Polymorphisms rs12593359 and rs11855560 were the predicted tag SNPs in our study; therefore, we believe that these variants may contribute the *RAD51* post-transcriptional regulation to some extent. Our results point to the mechanism underlying the effects of rs12593359 and rs11855560 on the regulation of *RAD51* expression and provide an explanation for the tumor susceptibility associated with these SNPs according to cancer research. It is possible that those genetic variants in *RAD51* 3′UTR may modulate its expression and the variants affecting *RAD51* miRNA-binding sites are associated with carcinogenesis.

In conclusion, the results of the present study suggest that the *RAD51* rs12593359 SNPs in DNA repair pathways may be utilized as predictive factors of the clinical outcome, and these SNPs may contribute to chemotherapy. In addition, our study may improve the understanding of the regulatory roles of miRNA variants of *RAD51* 3′UTR in its mRNA expression and the translation of pharmacogenetic predictors into clinical practice may lead to improved cancer treatment planning and outcome.

## Conclusion

In summary, *RAD51* variants have a strong influence on post-transcriptional regulation, and this finding highlights the importance of miRNA-mediated regulation of expression of cancer-associated genes, in particular, with respect to research on prognostic and diagnostic markers of malignancy. Besides, our findings should elucidate how the 3′UTR variants regulate *RAD51* activity and thus will pave the way to targeting of the *RAD51* pathway as a cancer treatment. Nonetheless, our data need to be validated by functional analysis of the mechanism underlying the association of *RAD51* transcriptional activity with variants in the 3′UTR.
